# Synergistic Effects between Lignin, Cellulose and Coal in the Co-Pyrolysis Process of Coal and Cotton Stalk

**DOI:** 10.3390/molecules28155708

**Published:** 2023-07-28

**Authors:** Xuehe Ding, Lihua Yan, Chang Guo, Dianzeng Jia, Nannan Guo, Luxiang Wang

**Affiliations:** State Key Laboratory of Chemistry and Utilization of Carbon-Based Energy Resources, College of Chemistry, Xinjiang University, Urumqi 830017, China; dxh428216850@163.com (X.D.); yanlihua0516@163.com (L.Y.); y1364523303@163.com (C.G.); jdz@xju.edu.cn (D.J.)

**Keywords:** coal, cotton stalk, cellulose, lignin, co-pyrolysis, synergistic effects

## Abstract

In this work, Qiqunahu (QQH) coal, cotton stalk, cellulose and lignin extracted from cotton stalk were selected as raw materials to study the effects of the co-pyrolysis of coal and cotton stalk. Online thermogravimetric mass spectrometry (TG-MS) was used to analyse mass loss and gas release characteristics during co-pyrolysis. The results reveal that the mixture of cotton stalk and coal can significantly enhance the reactivity of the blends and promote the formation of effective gas. The cellulose in the cotton stalk promotes the generation of H_2_ and CO_2_ during the co-pyrolysis of coal and cotton stalks. Lignin promotes the production of CH_4_ and CO_2_. Cellulose and lignin show an inhibitory effect on the precipitation of small molecular weight hydrocarbon gases during co-pyrolysis. This study provides a better understanding for the co-pyrolysis of biomass and coal.

## 1. Introduction

Energy demand continues to rise with the rapid development of the global economy [1,2]. Fossil fuels are a widely available energy resource, though this resource of energy production is associated with large amounts of greenhouse gas emissions, a driver of climate change [3,4,5]. Coal is still the largest raw material of power in China, which consumes billions of tons every year. However, as a kind of non-renewable energy, coal releases a lot of toxic gas (SO_2_, NO_X_, et al.) and other environmental pollutants during the combustion process. The government of China has proposed a target of becoming carbon neutral by 2060. Therefore, it is an urgent problem to cleanly and efficiently use coal resources.

As a renewable and eco-friendly energy source, biomasses are widely distributed on the Earth [6,7], which absorbs large amounts of CO_2_ as it grows. Meanwhile, the carbon contained in the biomass can be liberated in the gasification process. This indicates that the entire process of biomass growth and utilization is zero emission. However, biomass has low energy density, low gasification temperature and high transportation cost [8,9,10], which greatly limits the large-scale utilization of biomass.

The advantages of coal and biomass separate gasification can be fully exploited with mixed coal and biomass gasification technology, which is a promising energy utilization technology with high efficiency and clean conversion [11,12,13,14]. Co-pyrolysis is the first step in the co-gasification of biomass and coal, which has considerable influence on the following reactions [15]. Therefore, studies have been carried out in this area over the past decades. Co-pyrolysis includes the devolatilization of biomass and coal to form coke, tar and gases, resulting in solid-gases and gases-tar reactions [16,17,18]. The high hydrogen content indicates that biomass could act as a hydrogen donor in co-pyrolysis with coal [6,19,20]. Hence, co-pyrolysis of coal and biomass can not only pave the theoretical support for greater efficiency with better utilization of biomass and coal, but also reduce the activation energy of the reaction for co-pyrolysis of coal and biomass [21,22], which are conducive to industrializing and large-scale development. The whole process reduces polluting emissions and significantly improves utilization efficiency.

As the region with the most abundant coal and cotton stalk resources in China, Xinjiang has great potential in the gasification of coal and biomass. However, the mechanism of promoting or restraining effect in the co-pyrolysis process is still uncertain, and in-depth studies on co-pyrolysis are still lacking. Herein, the release behavior of small molecules during the co-pyrolysis of cotton stalk and coal were measured by the online thermogravimetric-mass spectrometer (TG-MS). The distribution of the gaseous products of cellulose, lignin, cotton stalk, QQH coal and their blend during co-pyrolysis were analyzed. This study is helpful to elucidate the interaction mechanism of coal and cotton stalk co-pyrolysis engineering, which provides the theoretical basis for large-scale industrial applications with high production efficiency. Co-pyrolysis of coal and cotton stalk can achieve dual benefits in environmental protection and economy, which provides a new direction for the practical significance for the future utilization of coal and cotton stalks.

## 2. Results and Discussion

### 2.1. Physicochemical Properties of Sample

**Figure 1:**
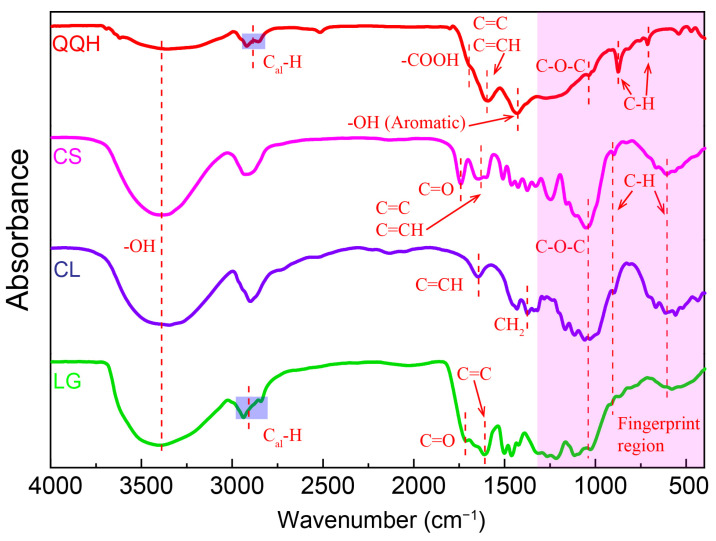
FT-IR spectra of four samples.

The FT-IR spectra in Figure 1 show that the chemical structures of the four raw materials are quite different [23,24,25]. Compared with the biomass-derived samples, raw coal shows weaker -OH, -C-O-C-, -COOH, -CH bands and stronger C=C, C=CH bonds, indicating the glucosidic bond of biomass-derived samples and the aromatic structure of raw coal, respectively. The FT-IR spectra of cellulose and lignin showed strong intensity of absorption peak at 3100–3700 cm^−1^, suggesting abundant surface hydroxyl groups. Moreover, the highest peaks of intensity of the out-of-plane deformation mode of -CH at 610 cm^−1^ and 870 cm^−1^ reveal the hydrogen in cotton stalks mainly comes from cellulose. Differently from cellulose, lignin and raw coal possess two absorption peaks at 3000–2800 cm^−1^ and an absorption peak at 1380 cm^−1^. This is mainly attributed to the abundant methylene in CL and rich -O-CH_3_ in the lignin and raw coal. The band at 1760 cm^−1^ caused by C=O stretching vibrations and the band at 1600 cm^−1^ derived from C=C vibration of lignin are much stronger than those of cellulose, suggesting the lactonic, carboxylic or anhydride, and aromatic ring groups in the cotton stalk mainly come from lignin. The highest peak at about 1400 cm^−1^ of raw coal suggests that the oxygen-containing functional groups of raw coal are mainly composed of phenolic hydroxyl groups.

### 2.2. Thermal Behavior Analysis of Sample

**Figure 2:**
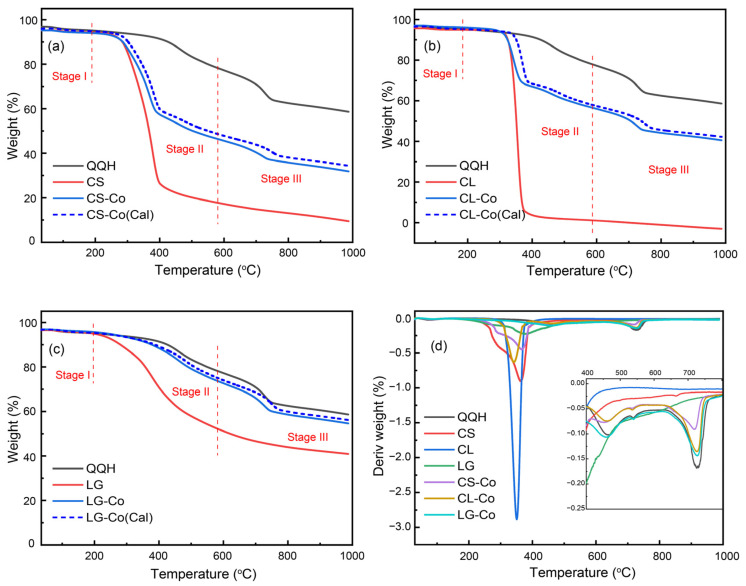
TG and DTG curves of sample pyrolysis: (**a**–**c**) TG; (**d**) DTG.

Figure 2 shows the TG and DTG curve of QQH, CS, CL, LG and their blends at a heating rate of 20 °C/min under an Ar atmosphere. As seen in the TG curves (Figure 2a–c), the whole pyrolysis is divided into three main stages. There is little weight loss in the first stage before 200 °C, which was caused by the evaporation of adsorbed water. The weight loss in the second stage is mainly derived from the thermal decomposition of the sample in the temperature range from 200−600 °C, with the peak approximately at 380−520 °C. The weight loss rate of CS and CL is 84.59% and 90.98% in the second stage, which are almost three times than the 20.18% and 24.10% of QQH coal and LG, respectively. This indicates that CS and CL release a lot of volatile gases during the pyrolysis process. Moreover, the pyrolysis process of CS and CL has been almost completed in the second stage. The pyrolysis temperature range of the third stage is range from 680−1000 °C. The weight loss peak of QQH appears in the temperature range between 715 °C and 900 °C. On the one hand, the relatively stable aromatic ring structure in coal occurs fracture reaction in this stage. On the other hand, the minerals in coal decompose at a high temperature, such as calcium carbonate (CaCO_3_). Figure 2d shows the DTG curves of all the samples. Compared to the CS, CL and LG, the initial pyrolysis temperature of the QQH coal is delayed, suggesting high temperatures are needed to disrupt the stable structure of raw coal. The addition of CS and CL make the weight loss peaks in the DTG curves of blends shift downward and increase the weight loss rate by 20.32% and 19.02% in the second stage. The blending of CL or CS with QQH can not only widen the devolatilization interval, but also increase the reactivity and the weight loss of QQH coal in the second stage. In contrast to the CL and CS, the TG and DTG curves of LG and coal blend have little obvious change with the original curves, indicating that the main reason for promoting coal pyrolysis is cellulose components in biomass precursors. By comparing the experimental curve and the calculated curve in Figure 2, the weight loss of the experimental curve is lower than that of the calculated curve, indicating the synergistic effect of coal and biomass during the co-pyrolysis process.

### 2.3. Evolution Curve of Low Molecular Weight Gases

#### 2.3.1. Separate Pyrolysis

**Figure 3:**
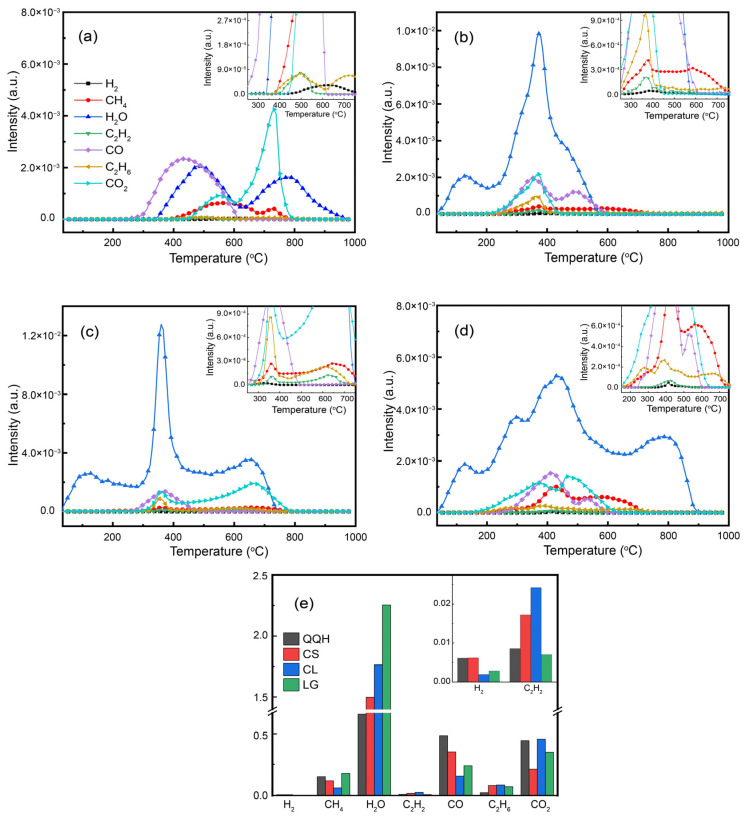
Gaseous evolution curves during pyrolysis: (**a**) QQH, (**b**) CS, (**c**) CL, (**d**) LG; and (**e**) gas yield.

The gas evolution curves during the pyrolysis of QQH, CS, CL and LG in TG-MS at a final temperature of 1000 °C are shown in Figure 3. As shown in Figure 3a, H_2_O, C_2_H_2_, CO and C_2_H_6_ gases begin to be released at lower temperatures, while H_2_, CH_4_ and CO_2_ gases are released at higher temperatures during the pyrolysis of coal. More H_2_O, CO and CO_2_ were produced because more oxygen-containing functional groups were present in the coal. The production of small gaseous hydrocarbons and H_2_ is less. Due to the low hydrogen content in coal, the hydrocarbon generation from the adipose side chain during pyrolysis is limited. On the other hand, H_2_ will participate in other reactions during pyrolysis. Hence, very little H_2_ can be released during pyrolysis of coal.

As for CS, CL and LG, their structure is less stable than that of coal, so gas molecules start to release at a lower temperature during pyrolysis. As shown in Figure 3b–d, H_2_O is released first, then CO, C_2_H_2_, C_2_H_6_ and CO_2_, followed by H_2_ and CH_4_. The low bond energy of ether and hydroxyl lead to a lower break temperature (200 °C), producing abundant H_2_O and CO_2_. Then, the aliphatic compounds begin to crack to form gaseous hydrocarbons when the pyrolysis temperature reaches 240 °C. In addition, the produced hydrocarbon gas will undergo secondary cracking, which results in hydrocarbon gas production near 600 °C. Then the alkyl chain starts to break at 300 °C, forming H_2_ and CH_4_. Figure 3e shows that the yields of H_2_O, CO, CO_2_ and small-molecule hydrocarbon gases during separate pyrolysis of biomass are significantly different from those of coal. For coal (QQH) and biomasses (CS, CL and LG), the composition and chemical structure are different, so the release characteristics of gas products are completely different. 

#### 2.3.2. Co-Pyrolysis of Coal and Biomass

**Figure 4:**
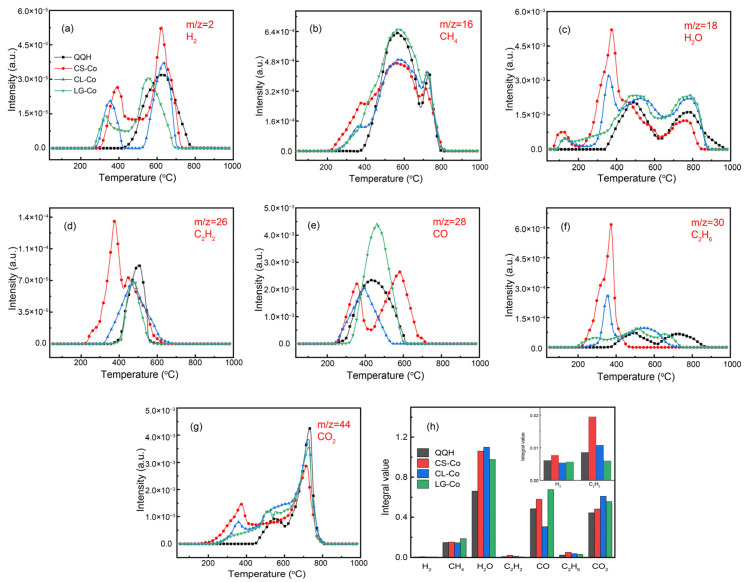
(**a**–**g**) Co-pyrolysis pyrolysis gas evolution curve; (**h**) gas yield.

Figure 4 shows the gas evolution curves of H_2_, CH_4_, H_2_O, C_2_H_2_, CO, C_2_H_6_ and CO_2_ in the co-pyrolysis process. Figure 4a shows that the release temperature range of sample H_2_ is 430−760 °C. There are three main sources of H_2_ in coal: (1) the combination of hydrogen free radicals generated after C-H bond breaking in the alkyl branch chain and the decomposition reaction of low molecular weight compounds at a low temperature [26]; (2) the dehydrogenation and condensation reactions of aromatic rings to obtain H_2_ at high temperatures; and (3) the secondary cracking reactions of large hydrocarbon molecules produced at high temperatures to produce partial H_2_ [27].

Different from the pure coal, the H_2_ release curves of blend show two peaks at 330−400 °C and 550−630 °C of biomass. Moreover, the H_2_ produced by the pyrolysis of CS-Co has a similar evolution curve with CL-Co and LG-Co, and the peaks are superpositions of CL-Co and LG-Co. These results reveal that H_2_ produced by the pyrolysis of cotton stalk is mainly derived from cellulose and lignin. The H_2_ release peak of CL-Co is stronger than that of LG-Co. This mainly is because most of the hydrogen in cellulose is stored in hydroxyl and ether structures, which are subject to cleavage reactions at lower temperatures. On the other hand, the hydrogen in lignin is mostly stored in alkyl or aromatic ring structures, and less hydrogen can be released at low temperatures (330−400 °C). At high pyrolysis temperature (550−630 °C), the water produced by the pyrolysis of CL-Co is higher than that of LG-Co, which is conducive to the reactions between water and coke to produce H_2_ [28]. In addition, H_2_ produced in the process of biomass pyrolysis will accelerate the cracking of long chain alkanes [29]. Combined with the peak area of H_2_ release curve in Figure 4h, the addition of CS can promote H_2_ production in the process of co-pyrolysis, but the increase of H_2_ production is small.

The evolution curve of CH_4_ during the pyrolysis of mixed samples was shown in Figure 4b. The temperature range of CH_4_ release is 400−780 °C for coal, and two distinct peaks occur at 543 °C and 712 °C. At lower temperatures, methyl groups attached to ether bonds and fat chains are first to break off and become methyl radicals, which link with the hydrogen radicals to produce CH_4_. The QQH coal produces more CH_4_ at a moderate temperature (400−600 °C), because of the high content of fat chains and -OCH_3_ in low-rank coal, as well as its easy fracture. At high temperature, the aromatic structure of coal begins to crack. The methyl group that is connected with the aromatic ring is split off by the heat to form the methyl radical, which forms CH_4_ in combination with the hydrogen radical [28]. Less CH_4_ is produced at higher temperatures, due to the relatively stable aromatic ring structure leading to less formation of methyl radicals.

Figure 4b shows that the release curves of CH_4_ in the mixed sample with CS, CL and LG ranges from 200−800 °C. A different QQH coal, CL-Co, appears at three acromial peaks at 350 °C, 568 °C and 725 °C due to the decomposition of light aromatics at a low temperature and the hydrocarbon-branched chains at a high temperature. LG-Co shows almost a similar curve with QQH, which is attributed to the similar chemical structure between LG and coal. Moreover, the CH_4_ yield of LG-Co is much more than that of CL-Co because the abundant (-O-CH_3_) will undergo a demethylation reaction after temperatures higher than 400 °C. The CH_4_ release pattern of CS-Co is superimposed by CL-Co and LG-Co, suggesting that the formation of CH_4_ is controlled by the interaction of cellulose, lignin and coal during the co-pyrosis process. Comparing the yield of the CH_4_ in Figure 4h, the addition of lignin increased CH_4_ production during co-pyrolysis, but the yield of CH_4_ decreased slightly after adding CL, which may be due to the large amount of hydrogen radicals reacting with hydroxyl radicals to generate abundant H_2_O during the pyrolysis of CL. Therefore, the increase of CH_4_ is limited in co-pyrolysis of coal and cotton stalk under the combined action of lignin and cellulose.

Figure 4c shows the evolution curve of H_2_O during the pyrolysis of mixed samples. The QQH coal began to release H_2_O at 354 °C, and the acromion appeared at 461 °C and 756 °C, respectively. When the temperature exceeds 354 °C, the carbonyl groups break off and react with hydrogen to form H_2_O, which shows an obvious peak at 461 °C. The peak at 756 °C is mainly attributed to the cracking of heavy hydrocarbons and the condensation of aromatic structures, which lead to the generation of H_2_O. The H_2_O release curves of the mixture of QQH coal and biomasses have a wide temperature range. The peaks near 100 °C are mainly caused by the release of adsorbed water from lignin and cellulose. At lower temperatures, the abundant aliphatic hydroxyl functional groups in the cellulose backbone are broken down to produce H_2_O, which corresponds to the release peak at 350 °C of CL-Co. However, the apparent peak in H_2_O release at 756 °C for LG-Co is due to more phenolic hydroxyl groups leaving the lignin. Comparing the yield of H_2_O in Figure 4h, the H_2_O yield of the blends during the co-pyrolysis is much more than that of QQH coal, which is advantageous for the gasification reaction and the water-gas conversion reaction [28]. The co-pyrolysis process of coal and CS produces more H_2_O than that of the co-pyrolysis process of coal mixed with CL or LG, which may be affected by the hemicellulose, another component of the CS. In addition, the alkali and alkaline earth metals in CS and coal catalyze the ring cracking reaction to generate carboxylic acid [30], thus increasing the output of H_2_O.

Figure 4d,f display the evolution curves of C_2_H_2_ and C_2_H_6_. Coal begins to release C_2_H_6_ at 380 °C and reaches its peak at 475 °C. The mixed samples with cellulose and lignin began to release C_2_H_6_ at 280 °C and 200 °C, respectively. At low temperatures, the fatty side chain breaks to produce carbon chains with lower molecular weight, and the vacant sites on the surface of these carbon chains bind hydrogen to produce C_2_H_6_ and C_2_H_2_. In the high temperature region, it is mainly obtained by cracking of heavy hydrocarbons. In addition, C_2_H_6_ is dehydrogenated to produce C_2_H_2_ with the gradual increase of temperature [31]. Comparing the yield of C_2_H_2_ and C_2_H_6_ in Figure 4h, the yield of C_2_H_2_ during co-pyrolysis of coal and CS increases greatly due to the positive effect of cellulose. The production of C_2_H_6_ increases slightly during co-pyrolysis, which is the result of the combined action of CL and LG.

Figure 4e shows the evolution curve of CO during the pyrolysis of the mixed samples. The release of CO for coal starts near 230 °C, and an obvious peak appears at 438 °C. The pyrolysis reactivity of the different functional groups in coal is: -OH > -O- > -C-H (on the fatty chain) > -C-H (on aromatic rings) > -C=O > -COOH > -CHO. In the early stages of pyrolysis, the active aldehyde groups break and reform to produce CO, and as the temperature continues to rise, the carboxylic groups undergo decarboxylation reaction to produce CO and CO_2_. When the temperature exceeds 400 °C, the methoxy in the coal and LG begins to break and combine with hydrogen radicals to produce CH_4_ and CO. Since lignin contains a large amount of methoxy, more CH_4_ and CO gas is produced during co-pyrolysis of lignin and coal. Moreover, the CO release of LG-Co was significantly different from that of pure LG, suggesting that the strong interaction between LG and coal is beneficial to the release of CO during the co-pyrolysis. On the contrary, the addition of cellulose is conducive to the production of CO_2_, which reduces the yield of CO. The CO release behavior of CS-Co is similar with the pure CS, which is caused by the combined action of CL and LG. Combined with the integrated peak areas of the CO release curves in Figure 4h, the addition of biomass feedstocks results in a substantial increase in CO production during co-pyrolysis. Comparing the CO yield during co-pyrolysis after the addition of cellulose and lignin, it is inferred that the lignin in CS plays a major role in the enhanced evolution of CO during co-pyrolysis with coal.

Figure 4g shows the evolution curve of CO_2_ during the pyrolysis of mixed samples. The process of CO_2_ release includes volatilization and carbonization stages. CO_2_ is mainly produced by the decomposition and reforming reaction of carboxyl and carbonyl groups during the volatilization phase. In the carbonization stage, the reforming reaction of C-O and carbonyl groups is the main reason for the generation of CO_2_. The temperature range of CO_2_ release during the pyrolysis of coal is 420−800 °C. The acromion appeared at 537 °C and 720 °C. Carboxyl and carbonyl groups on aliphatic and aromatic groups produce CO2 at low temperatures. At higher temperatures, CO_2_ is produced by the cracking of oxygen-containing heterocyclic compounds with ether structure, quinone structure and stable structure [32].

CL-Co and LG-Co began to release CO_2_ in a lower temperature region than pure QQH coal. This is induced by the low depolymerization temperature (near 200 °C) of cellulose and lignin. Only the lower strength ether bond in lignin will be C-O fracture. The acromion of CL-Co appeared at 360 °C, which was mainly caused by the breaking of carboxyl and ether bonds in CL. The acromion appeared at 512 °C in the mixed sample with lignin. On the one hand, C-C breaks are formed within alkyl chains in lignin. On the other hand, O-CH_3_ in the methoxy group of lignin is fractured. Therefore, the peak value of CO_2_ in the mixed sample with lignin at this stage is stronger than that of QQH coal pyrolysis alone. However, the CO_2_ yield in the high temperature region (600−800 °C) of the QQH coal is higher than that of mixed samples. This mainly results from the higher oxygen content groups in the QQH coal and the unstable oxygen-containing functional groups in CS breaking down at low temperatures. Combined with the peak area of the CO_2_ release curve in Figure 4h, the CO_2_ yield of mixed samples during pyrolysis is higher than that of QQH coal. LG and CL play a key role in the generation of CO_2_.

### 2.4. Evolution Curve of Benzene and Other Aromatic Compounds

#### 2.4.1. Separate Pyrolysis

**Figure 5:**
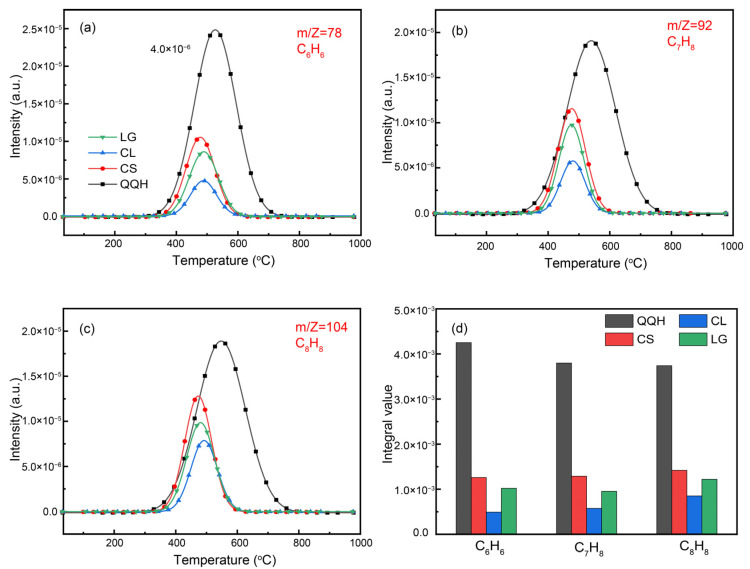
(**a**–**c**) Gaseous evolution curves during pyrolysis; (**d**) gas yield.

The evolution curves of benzene, toluene and styrene during sample pyrolysis are shown in Figure 5a–c. At about 380 °C, the aromatic compounds begin to form and reach a peak at 580 °C during the pyrolysis of coal. When the pyrolysis temperature is higher than 750 °C, no aromatic compounds are produced during coal pyrolysis. In the case of coal, the aromatic rings in the structure are connected by bridge bonds such as -CH_2_-, -O- and -S-. At the early stage of pyrolysis, the bridge bonds with weak stability become active and break, generating benzene ions or unsaturated structures with benzene rings, and then forming aromatic compounds through hydrogenation and other reactions. When the pyrolysis temperature is 450−650 °C, most of the bridge bonds are broken and more unsaturated structures of benzene rings are formed. When the pyrolysis temperature is higher than 650 °C, the secondary reaction of semi-coke will also produce aromatic compounds.

Compared with coal, the yield of aromatic compounds during pyrolysis of biomass samples is less (Figure 5d). This is because of the lower aromaticity of biomass, fewer aromatic rings in the structure and relatively few aromatic compounds being generated by pyrolysis. In addition, the yield of aromatic compounds in the pyrolysis of lignin is higher than that of cellulose due to the developed aromatic structure in molecular chains of lignin.

#### 2.4.2. Co-Pyrolysis of Coal and Biomass

**Figure 6:**
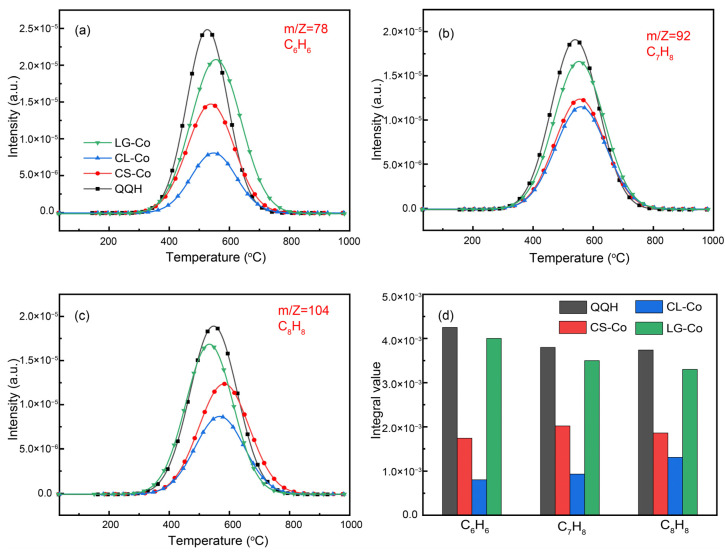
(**a**–**c**) Co-pyrolysis pyrolysis gas evolution curve; (**d**) gas yield.

The evolution curve of benzene, toluene and styrene in the mixed pyrolysis process is shown in Figure 6a–c. During co-pyrolysis of cotton stalk and coal, benzene and other aromatic compounds begin to be released at 350 °C, reach a peak at 550 °C and end at 750 °C. Compared with the separate pyrolysis of coal, there is no significant change, which indicates that the synergistic effect of cotton stalk and coal co-pyrolysis has little effect on the release of benzene and other aromatic compounds. In addition, the yield of benzene and other aromatic compounds is the smallest when cellulose is mixed with coal pyrolysis (Figure 6d). This may be because the fragments formed by the cracking of cellulose at the early stage of pyrolysis combine with the aromatic structure in coal, which increases the molecular weight of the unsaturated structure-containing benzene ring formed by the fracture of bridge bonds in coal, and thus deposits semi-coke in coal coke. Hence, the released content of benzene and other aromatic compounds is significantly decreased.

### 2.5. Synergistic Effect

**Figure 7:**
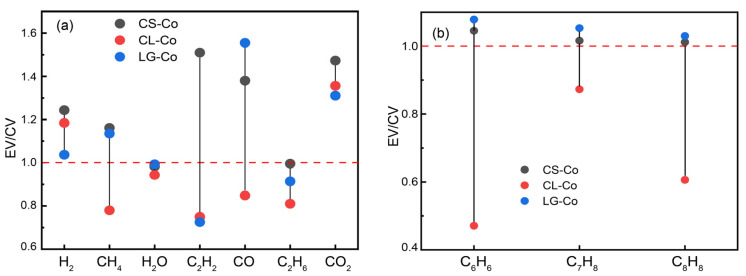
Ratio of experimental to calculated values. (**a**) low molecular weight gases; (**b**) benzene and other aromatic compounds.

The ratio of experimental to calculated values of the yield is shown in Figure 7. As shown in Figure 7a, the synergistic effect during the co-pyrolysis of coal and cotton stalk promotes the release of H_2_, CH_4_, C_2_H_2_, CO and CO_2_. The promotion of CO_2_ during co-pyrolysis is similar for cellulose and lignin. The promotion effect of cellulose on H_2_ during co-pyrolysis was stronger than that of lignin, and the promotion effect of lignin on CH_4_ and CO was stronger than that of cellulose. In addition, CS promotes C_2_H_2_ during co-pyrolysis with coal, but cellulose and lignin inhibit it during co-pyrolysis with coal. This suggests that the other components of CS promote the release of C_2_H_2_ and the complex interactions between the components of the CS during co-pyrolysis.

As seen in Figure 7b, the EV/CV values of C_6_H_6_, C_7_H_8_ and C_8_H_8_ are greater than 1, indicating that the synergistic effect during co-pyrolysis of coal and cotton stalks promotes the release of benzene and other aromatic compounds. Moreover, the EV/CV > 1 of C_6_H_6_, C_7_H_8_ and C_8_H_8_ during co-pyrolysis of lignin and coal, while the EV/CV < 1 of C_6_H_6_, C_7_H_8_ and C_8_H_8_ during co-pyrolysis of cellulose and coal. It can be determined that cellulose in cotton stalks inhibits the release of benzene and other aromatic compounds, while lignin promotes their release.

**Figure 8:**
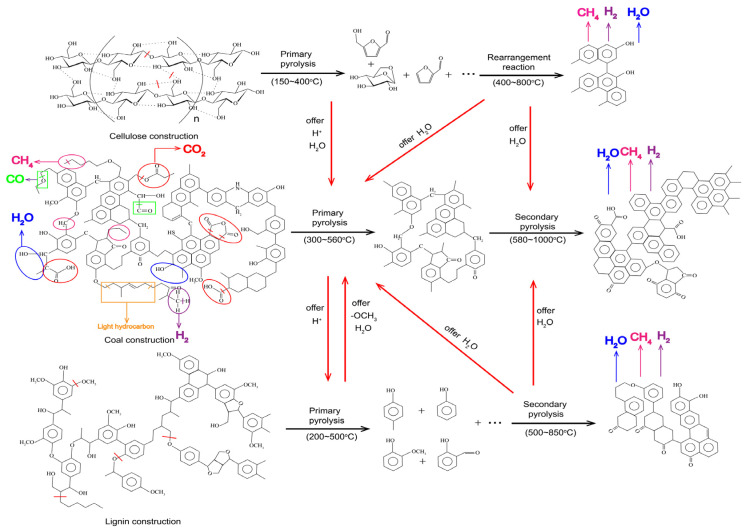
Diagram of the synergistic mechanism of cellulose and lignin with coal pyrolysis.

The synergistic interaction between cellulose, lignin and coal is shown in Figure 8. Abundant fragmented structures are formed at the beginning during cellulose pyrolysis [33,34,35]. In addition, the H^+^, H_2_O and -OCH_3_ produced by the cleavage of cellulose and lignin participate in the formation of CO and CH_4_ in the coal, as in Equations (1)–(3). The large amount of water produced by cellulose at the beginning of pyrolysis facilitates the reaction in Equation (4), resulting in a reduction of CH_4_ production.

-CH_2_- + H^+^ → CH_4_ (1)

-CH_3_ + H^+^ → CH_4_ (2)

-O-CH_3_ → O·+ -CH_3_ + H+ → CO+ CH_4_ (3)

H_2_O + CH_4_ → CO_2_ + CO + 2H_2_ (4)

Char + H_2_O → CO + CO_2_ + H_2_ (5)

As the pyrolysis temperature gradually increases, hydrogenation of the more stable hydroxyl groups in cellulose and lignin occurs, producing water and facilitating the reaction in Equation (4). As the temperature continues to increase, the Char from the primary pyrolysis of cellulose and lignin undergoes polymerization to produce H_2_O, CH_4_ and H_2_. The produced H_2_O will react with the char formed from the primary pyrolysis of the coal (Equation (5)), which further promotes the production of CO, CO_2_ and H_2_ in the high-temperature region.

## 3. Materials and Methods

### 3.1. Sample Collection and Blend Preparation

The raw materials selected for this study were cellulose, lignin, cotton stalk and Qiquanhu bituminous coal (QQH). The QQH coal and cotton stalk are grounded to 200 mesh and 80 mesh, respectively. Cellulose and lignin were used as the biomass model components, which are extracted from cotton stalks by the nitric acid-ethanol methods [36,37] and Klason lignin method [38], respectively. According to the methods in previous works, the cotton stalk contains 20.3 wt% lignin and 37.4 wt% cellulose. In a typical process, 0.374 g cellulose, 0.203 g of lignin and 1 g of cotton stalk were mixed with 1 g QQH coal to form co-pyrolysis precursors, which were denoted as CL-Co, LG-Co and CS-Co, respectively. The proximate and ultimate analysis of cellulose, lignin and cotton stalk were performed using SDTGA5000a, SDCHN536 and SDS820. Proximate analysis and ultimate analysis of the raw materials are given in Table 1. The content of inorganic atoms in coal and cotton stalk is shown in Table 2.

### 3.2. Methods

The online thermogravimetric mass spectrometer (TG-MS) was used in this experiment. Temperature rises from room temperature to 1000 °C at a rate of 20 °C/min. The test range and maximum operating temperature of the mass spectrometer are 0−300 amu and 300 °C, respectively. Ar was used as the carrier, with a flow rate of 150 mL/min.

The difference between the experimental value (EV) and calculated values (CV) of the gas yield was used to assess the synergistic interaction of QQH coal with CS, CL and LG. The calculated values were obtained by the following equation:

CV = EV_coal_ × *w* + EV_biomass_ × (1 − *w*)

CV represents the calculated gas yield for the blends. EV_coal_ and EV_biomass_ represent the experimental gas yield for the pyrolysis of coal and biomass samples alone, respectively. *w* represents the mass ratio of coal in the blends.

## 4. Conclusions

In summary, the addition of cotton stalks to coal promotes coal pyrolysis and leads to an increase in volatile gas production. Lignin and cellulose play different roles during the co-pyrolysis of cotton stalks and coal. According to the online TG-MS results, the addition of cotton stalk in QQH coal can significantly enhance the reactivity of the blends and promote the formation of effective gas. Cellulose in cotton stalk contains more hydrogen and hydroxyl, which leads to more H_2_ and H_2_O production during co-pyrolysis. At 400 °C, the lignin in the cotton stalk began to crack and promoted the production of CH_4_ and CO_2_ during co-pyrolysis. The other components of the cotton stalk promote the production of C_2_H_2_ during co-pyrolysis, and have little effect on C_2_H_6_ production. In this study, the specific content of components in cotton stalks was determined, and the cellulose and lignin were co-pyrolyzed with coal according to their content in cotton stalks. The effect of the components in the cotton stalks on the co-pyrolysis was analyzed semi-quantitatively. This study has an important reference value for clean and efficient utilization of biomass and coal, and provides helpful guiding significance for determining the interaction mechanism of various mixed samples in the co-pyrolysis process.

## Figures and Tables

**Table 1:** Proximate and ultimate analyses of samples.

Samples	Proximate Analysis, ad, wt%				Ultimate Analysis, d, wt%				
	M	VM	FC	A	C	H	S	O ^a^	N
QQH	3.10	43.09	40.16	13.65	62.24	3.41	1.09	18.84	0.77
CS	4.30	82.39	12.52	0.79	47.37	6.41	0.05	44.87	0.51
CL	3.69	90.03	6.24	0.04	43.04	6.51	0.03	49.68	0.70
LG	3.80	53.51	39.02	3.67	59.45	5.23	5.41	25.62	0.62

Note: M—moisture; VM—volatile; FC—fixed carbon; A—ash; ad—air-dry basis; d—dry basis. ^a^ Calculated by difference.

**Table 2:** The content of inorganic atoms in samples.

Sample	mg/kg					
	K	Ca	Na	Mg	Al	Fe
QQH	612	20,314	4743	6587	1959	426
CS	902	1406	940	479	465	665

## Data Availability

Data is contained within the article.

## Sample Availability

Samples of the compounds are available from the authors.
